# Impact of COVID-19 on Surgical Volume: Single-Center Experience from Addis Ababa, Ethiopia

**DOI:** 10.4314/ejhs.v32i1.5

**Published:** 2022-01

**Authors:** Yonas Ademe, Abraham Genetu, Tsegazeab Laeke, Mulat Taye, Abebe Bekele

**Affiliations:** 1 Addis Ababa University, College of Health Sciences, School of Medicine, Department of Surgery Addis Ababa, Ethiopia; 2 Professor of Surgery, University of Global Health Equity, School of Medicine Kigali Heights, Plot 772, KG 7 AVE

**Keywords:** COVID-19, pandemic, operation, surgical volume

## Abstract

**Background:**

The COVID-19 pandemic has caused substantial disruptions to surgical-care delivery mainly due to diversion of available resources from surgical to COVID-19 care, reduced flow of patients, supply-chain interruptions and social distancing and restriction measures. The purpose of this study was to understand the impact of the pandemic on surgical volume in our hospital.

**Methods:**

A descriptive cross-sectional study was done at Tikur Anbessa Specialized Hospital. A 2- year data was collected from March 2019 up to March 2021 from the operation theatre registration books. The data registry at the admission records office was also reviewed to extract the number pf patients on the elective surgery waiting list. Data were recorded, analyzed and reported using SPSS software package 26.

**Results:**

The findings showed that there was a significant drop in surgical volume during the COVID-19 era. Surgical volume has dropped by 19% for emergency and by 32% for elective surgeries. COVID-19 test positivity of patients was identified as the single most important reason for elective operation cancellation during the first wave of the pandemic, contributing to as high as 85% of the reasons.

**Conclusion:**

The outcome of our study showed that COVID-19 has adversely affected elective and emergency surgical volume in our institution. This has also led to a dramatic increase in the surgical waiting list load. We recommend immediate surgical systems strengthening measures to re-build the surgical care ecosystem significantly affected by COVID-19. Surgical and anesthesia systems strengthening should be an integral part of pandemic preparedness and management.

## Introduction

The novel coronavirus (SARS-CoV-2) was first documented on 31 December 2019 in Wuhan, China followed by the first death on 11 January 2020. From there, the virus quickly spread at unprecedented rates across the globe (1–4). On 30 January 2020, the WHO declared a global state of emergency and named the novel coronavirus Covid-19 on 11 February as the virus spread across the world from China to South Korea and Iran, and then Italy. On March 28, the first case of COVID-19 was reported in Ethiopia. During the first wave of the pandemic in the country, between April and August of 2020, the Federal Ministry of Health of Ethiopia reported thousands of people infected with the virus with alarming number of mortalities. The pandemic of COVID-19 is disrupting global health, social welfare and the economy in a proportion unparalleled in modern history.

Several authors have reported on the impact of the pandemic on health care. A report from Australia showed that, during the 4 months after COVID-19, primary care face-to-face consultations decreased by 22.1%, breast screening activity by 51.5%, ambulance incidents by 7.2%, emergency department visits by 13.9%, public hospital inpatient episodes by 14.3%, and public hospital planned surgical activity by 32.6% (5).

Surgical delivery, owing to its cross-cutting nature and synergistic effects on health systems at large, has been adversely affected by the pandemic. In the current pandemic, rather than mobilizing surgical resources for surgical conditions in need, the current demand for ventilators, hospital space and personnel is depriving surgical capacity to a point where essential surgical delivery is strained in multiple regions, irrespective of their economic classification. This is having an immediate and long-term effect on millions of patients with surgical conditions worldwide (6). All over the world, millions of cases were cancelled, resources were diverted to COVID-19 care, theatres were converted to COVID-19 ICUs, campaigns were cancelled, and nurses and doctors were re-allocated from surgical to COVID-19 care (6,7,8,9,10). Surgical education has also been disrupted with several residency programs delayed (11–13).

Studies have reported the dangers associated with operating patients infected with SARS-COV2. These risks should be balanced against the risks of delaying surgery in individual patients. Before the SARS-CoV-2 pandemic, high-quality, multinational observational studies established overall baseline rates of postoperative pulmonary complications (up to 10%) and subsequent mortality (up to 3%) after surgery (14–16). In contrast, a recently published international, multicenter, cohort study reported that postoperative pulmonary complications occur in half of patients with perioperative SARS-CoV-2 infection and are associated with high overall mortality (30–day mortality of 23.8%) (17). Part of the reason for disrupted surgical services during the COVID-19 era originated from the risks to health workers associated with the virus. As PPE constraints grow, the number of infections of health care workers grows accordingly. It is to be expected that these developments have even more grave consequences in LMICs, such as ours, where the luxury does not exist to pull the limited surgical workforce out of the operating rooms whilst maintaining emergency and essential surgical care delivery, and where screening, testing, and PPE is even less accessible.

At the surgical department of our institution, we were prioritizing patients with malignancies and obstructive pathologies. Every elective patient had to have a negative SARS COV 2 test, which sometimes took up to two weeks. Numerous precautionary measures were also taken to decrease spread of the infection among staff. In light of these considerations however, the overall surgical activities of the hospital were presumably compromised. To reflect this issue, the authors report on the effects of the COVID-19 pandemic on surgical activities at a tertiary hospital.

## Methods and Materials

This descriptive cross-sectional study was done at Tikur Anbessa Specialized Hospital. The hospital is the teaching hospital of Addis Ababa University, College of Health sciences, and it is the main tertiary referral hospital for the country. It has a 550 bed-capacity, out of which close to 260 is dedicated to surgical care.

A 2-year data was collected from March 2019 up to March 2021 from the operation theatre registration books for five major surgical divisions: General and vascular surgery, Cardiothoracic surgery, Pediatrics surgery, Neurosurgery, and Urology. The specific study period was chosen to compare the surgical volume during the year before and during the COVID-19 pandemic. Data elements collected included monthly and total number of cases operated, type of procedures performed, and whether the case was elective or emergency. Data was also collected from the hospital's admission records office (Liaison office) to record the patterns of patients on the waiting list before and after the COVID-19 pandemic.

Raw data was analyzed using nonparametric statistical methods with the help of SPSS software package 26. Descriptive statistics formed the mainstay of the statistical analysis. The study was done after seeking permission from the hospital authorities and a written ethical clearance letter was obtained from the departmental research ethics committee.

**The following operational definitions are used**

**Emergent surgical cases**: patients likely to have survivorship compromised if surgery not performed within next few hours (18)

**Urgent surgical cases**: patients likely to have survivorship compromised if surgery is not performed within next few days (18)

**Semi-urgent surgical cases**: patients likely to have survivorship compromised if surgery is not performed within next three months (18)

**Non-urgent surgical cases**: patients whose survivorship is unlikely to be affected if surgery is not performed (18)

**Elective operation**: an operation that is planned in advance, rather than one that's done in an emergency situation. (18)

**Personal Protective equipment**: is a list of protective equipment used to prevent airborne infection (such as SARS CoV-2 virus) including cap, face shield, surgical mask, goggle, gown, apron, boots, boor cover, and surgical gloves

## Results

**Figure 1:**
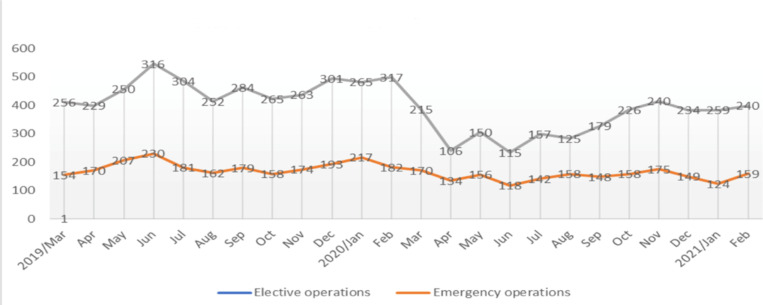
Patterns of monthly total number of elective and emergency operations

**Surgical volume**: The results of the study show that there was a significant drop in operations performed by all surgical divisions during the period after COVID-19 was first reported in the country. A total of 5509 (2,207 emergencies and 3,302 elective surgeries) were performed over a period of one year before COVID-19 [March 2019 – February 2020], while the total volume dropped to 4,037 (1,791 and 2,246 respectively) during the COVID-19 epidemic [March 2020 – February 2021]. The drop in Surgical volume was 19% for emergency and 32% for elective procedures. The maximum monthly elective surgical activity was 317 surgeries during the pre-COVID-19 period as opposed to 106 surgeries during the COVID-19 period. Operative volume in all surgical divisions was comparably affected. Routine activity levels seem to be recovering by September 2020. They, however, had not yet returned to ‘normal’. Figure 1 shows patterns of monthly total number of elective and emergency operations.

**Figure 2:**
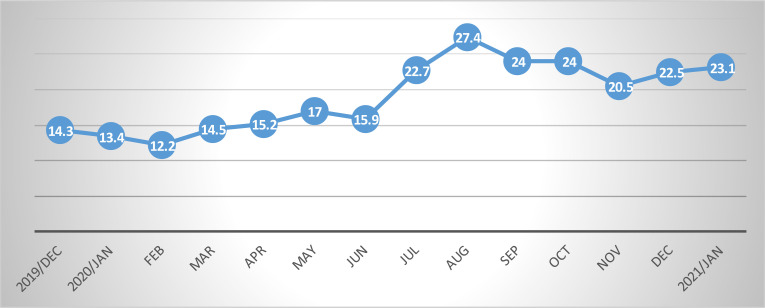
Pattern of monthly elective operation cancellation rates

**Figure 3:**
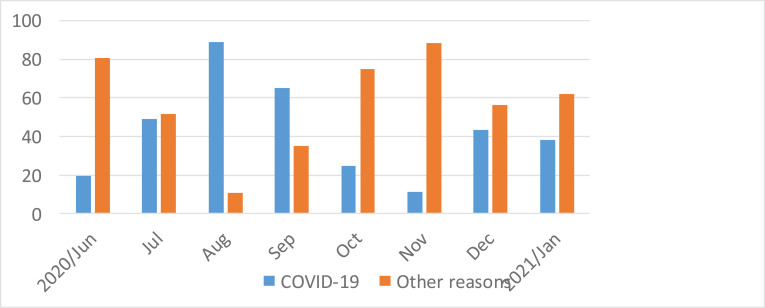
Reasons for elective operation cancellation

**Elective operation cancellation rates**: The weekly elective operation cancellation rate increased significantly during the COVID-19 era and COVID-19 positivity of patients was identified as the single most important reason for case cancellations, contributing to as high as 85% of the reasons for elective operation cancellations (Figure 3). Pre-COVID-19 cancellation rate data could be retrieved only for the 4 months prior to the first case of COVID-19 in the country and from this the average elective operation cancellation rate was calculated to be 13.6%. During COVID-19, it can be seen that this has showed a dramatic increase to 21.3% and the highest cancellation rate of 27.4% was recorded on the 5^th^ month after COVID-19. Overall, within the included period for which it was possible to retrieve data regarding reasons for elective operation cancellation, COVID-19 related reasons accounted for 137 (40.5%) of the cancellations. Other common reasons for elective operation cancellation identified were shortage of time 17 (21%), incomplete preoperative patient preparation 37 (11%), and lack of water and/or drapes 34 (10%). Figure 2 shows the pattern of monthly elective operation cancellation rates during the study period, Table 2 depicts identified reasons for elective operation cancellations, and Figure 3 shows the pattern of COVID-19's shares as a reason for elective operation cancellations.

**Table 2:** Reasons for elective operation cancellation (June, 2020 – January, 2021)

Reason for cancellation	Number (%)
COVID-19 related	137 (40.5)
Shortage of time	71 (21)
Incomplete pre-operative patient preparation (COVID-19 un-related)	37 (11)
Lack of water/drapes	34 (10)
Lack of blood	20 (6)
Lack of oxygen	17 (5)
Lack of ICU beds	13 (4)
Lack of instruments	8 (2.5)
Total	338 (100)

**Surgical waiting list and waiting time for elective surgery**: The surgical waiting list data for the 5 surgical divisions that show the burden of elective cases indicate that, as of the last day of data collection for this study, there were a total of 2,439 patients waiting for surgery, of which 993 patients (41% of total) were added to the waiting list during the 1-year period after COVID-19. Among the total number of patients on the waiting list 1,047 were general and vascular, 848 urological, 84 cardiothoracic, 85 neurosurgical, and 375 pediatrics surgical. Although 703 (28.8%) of the patients on the waiting list had non-urgent surgical cases, it was found that the majority 1261 (51.7%) had semi-urgent surgical cases and a significant proportion 475 (19.5%) were found to be waiting for an urgent surgical intervention. Before the COVID-19 era, the average waiting time for semi-urgent cases was 112±35 days and it was 41±17 days for urgent cases. After COVID-19, this has shown a dramatic increase and it was 298±76 days and 108±42 days for semi-urgent and urgent cases respectively. Table 1 shows the surgical waiting list data for the five divisions of surgery included in this study.

**Table 1:** Number of patients waiting for elective operation at TASH.

Surgery division	Total number of patients waiting for surgery		
General and Vascular Surgery (total)	1,047	Non-urgent	453
		Semi-urgent	462
		Urgent	132
Endocrine surgery	229		
Colorectal surgery	192		
Hepatobiliary surgery	205		
Upper GI surgery	19		
Vascular surgery	233		
Miscellaneous general surgery	179		
Urology	848	Non-urgent	153
		Semi-urgent	485
		Urgent	210
Neurosurgery	85	Non-urgent	15
		Semi-urgent	43
		Urgent	27
Pediatrics surgery	375	Non-urgent	69
		Semi-urgent	218
		Urgent	88
Cardiothoracic surgery	84	Non-urgent	13
		Semi-urgent	53
		Urgent	18
Total	2,439	Non-urgent	703
		Semi-urgent	1261
		Urgent	475

## Discussion

The ongoing COVID-19 pandemic is having a serious collateral negative effect on delivery of surgical care to millions of patients. Little has been known about pandemic response and its effects on other services, including delivery of surgical care. It has now become evident that the collateral effect from near-universal disruption and cancellation of surgical services has emerged. Although the seasonal disruption of regular surgical care and the occasional cancellation of surgery is not new to most healthcare systems, the current pandemic has shown an unprecedented implication for surgical services and patients with surgical disease (9).

Shortly after COVID-19 was first reported in Ethiopia, the majority of public hospitals have stopped operating on all elective surgical cases. Non-urgent, non-cancer procedures were fully cancelled, and staff were reallocated to assist in emergency COVID-19 care. This is consistent with the steps taken by various countries and recommended by several authorities (18). One article advocated that truly elective operations should be postponed preserving PPE, staff and facility capacity as important resources during a surge response (19).

Our study showed that there was a 32% decline in elective surgical volume during the COVID-19 period. This decline was most prominent during the first wave of the pandemic in Ethiopia which was between April and August of 2020. The elective surgical volume decline was consistent with several other reports since most health-care systems responded to the pandemic by cancelling or delaying elective surgical procedures mainly to protect patients and health-care workers from un-necessary exposure to the infection. Delaying elective surgeries was also aimed to preserve resources and mitigate the realities of meeting a surge in demand. A report from Nigeria reported a 68% decline in elective surgeries over the course of during the first 5 months of the pandemic (10). Another study from Australia found a decline in public hospital planned surgical activity by 32.6% (5). Similarly, a study from Italy reported a 75% decline in elective surgeries, a 30% decline in emergency surgeries and an overall 68% decrease in all operative activities (11).

In our study, it was found out that there was a reduction in emergency operations by 19% during the COVID-19 era compared to the one year before COVID-19. A Nigerian study showed a steady decline by about 41% in emergency surgeries during the first 5 months of the pandemic (10). These data show the need to adopt consensus guidelines tailored to maintain emergency services in LMICs during this pandemic. Follow-up studies may also evaluate the case mix of emergency surgeries during the period of decline and determine if certain categories of diseases were more affected than others. Additionally, the observed and unexpected reduction in emergency surgical procedures highlights the need to prioritize access to care for patients with emergency surgical conditions as part of pandemic planning.

A global predictive modelling on elective surgery cancellations estimated that in 190 countries 28,404,603 operations would be cancelled or postponed during the peak 12 weeks of disruption due to COVID-19 (2,367,050 operations per week). It estimated most would be operations for benign disease (90.2%). The predictive modeling estimated weekly total elective operation cancellations for Ethiopia to be 1124 elective operations, which would be close to 15,000 procedures in the three months cited (21). In our study, during the first few months of the pandemic, monthly cancellation rates exceeded 20% and the most important reason was identified to be COVID-19 infection and factors related to it.

In the global predictive modelling, it was also estimated that if countries increased their normal surgical volume by 20% after the pandemic, it would take a median of 45 weeks to clear the backlog of operations resulting from COVID-19 disruption thereby prolonging the waiting time for elective operations (21). In our study, the suspension of most elective surgeries was found to have an impact on elective case waiting lists. As of the last day of data collection, nearly 2,500 patients were waiting for surgery and a nearly half of these were added to the list during the COVID-19 era. This clearly has significantly increased the waiting time for elective operations (Pre- vs COVID-19: 112±35 days vs 298±76 days for semi-urgent cases and 41±17 days vs 108±42 days for urgent cases). García-Rojo E et al reported a mean time on elective surgical waiting list of 97.33±55.47 days after the COVID-19 era. In their study, priority 1 patients, who normally should undergo surgery within 30 days, were reported to be on the waiting list for a mean time of 60.51±20.14 days (22). In comparison to this study, results from our study showed that the absolute duration of waiting time for elective surgery was higher but the relative increment during the COVID-19 era was comparable.

In conclusion, provision of surgical services will continue to be an essential and integral component of healthcare system throughout the pandemic, and surgical systems will have to adapt to a continuously changing situation. The pandemic has also shown us that earlier strengthening of our surgical care system could have prepared us for the pandemic. The results of our study provide important information to highlight the impact of COVID-19 on elective and emergency surgery in TASH in particular and Ethiopia in general. It can be used as a benchmark for planning and resource prioritization during the transition phase, ensuring quality standards and safety of patients and staff. Ethiopia needs to design a nation-wide strategy to address the serious negative consequences of the pandemic. These could include surgical campaigns to reduce the waiting list for elective surgeries. However, the main message remains to be strengthening the national surgical and anesthesia care in the country.

We believe this study will be of significant importance in providing basic information on the changes in pattern of surgical activities in the COVID-19 setting. However, the utilization of secondary data sources in the background of poor data management system in the Hospital restricted the authors from including additional variables in the study. Incomplete documentation and as a result poor retrieval of archived data has led to asymmetric comparisons in some parameters between the two years included in the study. For example, data regarding cancellation rates could not be retrieved for the first six months of the pre-COVID-19 year. Similarly, data regarding reasons for elective operation cancellation could only be retrieved for the time period between June, 2020 and January, 2021.
